# Type A Behaviour Pattern and Health Behaviour of Polish Nurses

**DOI:** 10.3390/ijerph19116358

**Published:** 2022-05-24

**Authors:** Lucyna Gieniusz-Wojczyk, Józefa Dąbek, Halina Kulik

**Affiliations:** 1 Department of Propaedeutics of Nursing, Faculty of Health Sciences in Katowice, Medical University of Silesia, 40-027 Katowice, Poland; hbkulik@gmail.com; 2 Department of Cardiology, Faculty of Health Sciences in Katowice, Medical University of Silesia, 40-635 Katowice, Poland; jdabek@sum.edu.pl

**Keywords:** nurses, lifestyle, type A personality, health behaviours

## Abstract

“Coronary prone behaviour pattern” refers to a way of coping with environmental stressors, otherwise known as type A behaviour patterns. Stress, unlimited working hours, and the shift system are conducive to an “unhealthy life style”, conducted by nurses. The aim of the study was to assess the “coronary prone behaviour pattern”, taking into account health behaviour and work performed by Polish nurses. Method: This was a descriptive study conducted from June 2017 to May 2018 among nurses (N = 1080) working primary care or in training facilities in Silesia, Poland. Data were acquired through a series of questionnaires and are presented as descriptive statistics. Results: The “coronary prone behaviour pattern” (type A behaviour) was manifested by 333 (30.8%) nurses, type B by 272 (25.2%). The “coronary prone behaviour pattern” respondents showed a risk of developing a problem with alcohol (*p* = 0.003) less frequently compared with other respondents. In addition, nurses with the abovementioned behaviour pattern ate better (M = 16.66; SD = 6.11) compared with those with the type B behaviour pattern (M = 15.49; SD = 6.52). In terms of mental and physical wellbeing, people with type A behavioural patterns had significantly (*p* < 0.001) better mental and physical wellbeing and, on average, better results in coping with stress compared with other behavioural patterns of nurses. Conclusion: The occurrence of the “coronary prone behaviour pattern” was associated with the health-promoting behaviours of nurses being the subject of the analysis, i.e., better mental and physical wellbeing, better ability to cope with stress, and a lower risk of problems with alcohol and proper nutrition.

## 1. Introduction

The current global shortage of staff in the nursing profession is becoming a global challenge [1]. Considering the growing demand for care (ageing population) and the ageing of nursing personnel, we need to consider the implementation of strategies aiming to improve their mental and physical health. Heart disease has been the main cause of death worldwide for 20 years. However, today, the number of deaths caused by heart disease has increased from more than 2 million in 2000 to almost 9 million in 2019. Despite the progress in diagnosis and treatment, the role and importance of modifiable risk factors related to lifestyle (smoking, alcohol abuse, improper nutrition, low physical activity, and coping with stress) in the development and progression of cardiovascular diseases has not changed. In addition, personality traits are becoming more and more important in the occurrence of cardiovascular diseases. Taking them into account in prevention may significantly reduce morbidity and mortality, as well as reduce the costs borne by the state related to the treatment and absenteeism of those affected by them [2].

The previous research indicates that improper health behaviours among nurses are related to professional challenges, including high mental and physical strain, long working hours, and shift work [3,4,5,6,7,8]. During the last two decades, type A behaviour patterns and their opposite, type B, have been subjects of interest among scholars dealing with business, medicine, and psychology [9,10]. The type A behaviour pattern, defined as the “coronary prone behaviour pattern”, was introduced in 1959 by two Californian cardiologists, M. Friedman and R. Rosenman. The authors suggest that a type A behaviour pattern is not a specific personality profile nor solely a set of typical reactions, but a specific way of dealing with environmental stressors [11]. The research suggests that people who feel strong time pressure, who have type A behaviour patterns, are more prone to the development of coronary disease than more relaxed people, the type B behaviour pattern [12]. People with type A behaviour patterns experience stress more often and are characterised by the increased reactivity of the cardiovascular system, manifested by significantly higher arterial pressure and heart rate in response to a working stressor. They may suffer from a gradual and irreversible deformation of the myocardium and the accelerated development of atherosclerotic lesions in excessively loaded artery walls [13]. Suppression of hostility and remaining in a state of increased tension causes the consolidation of psychophysical reaction patterns, harmful to health. However, after a period of nearly sixty years of conceptualization and research related to the assumed dependence between type A behaviour patterns and cardiovascular diseases, it is difficult to identify a clear-cut model clarifying the above relation [14,15]. Some authors suggest that the type A behaviour pattern is most likely a risk factor of diseases only among several people, interacting with certain properties of temper (that is, their type of nervous system). Cooccurring with activity, as a feature of temper, does not constitute a risk factor. However, a relation of high hyperreactivity of temper with the type A behaviour pattern results in experiencing negative emotions, which is adverse for health [16]. Currently, the occurrence of cardiovascular diseases is combined with specific elements belonging to type A behaviour patterns, that is, anger and hostility, which were indicated by ESC and the Polish Cardiac Society as psychosocial risk factors for cardiovascular diseases [17].

The aim of the study was to assess the “coronary prone behaviour pattern”, taking into account health behaviour and work performed by Polish nurses.

The main research questions in this study concerned the following issues:

– What is the A/B personality pattern among the nurses who are the subjects of the study?
– Do the A/B personality patterns differentiate nurses in terms of health-related behaviours?

## 2. Materials and Methods

### 2.1. Design, Setting, and Participants

This was a descriptive, multicentre study, conducted from June 2017 to May 2018 in the following healthcare units (these were multidisciplinary, public centres) and training centres for nurses in Silesia: Regional Specialised Hospital No. 4 in Bytom; University Clinical Center, prof. K. Gibiński of the Medical University of Silesia in Katowice; Medical University of Silesia; Training Centre for Nurses and Midwives in Łagiewniki; Post-graduate Training Centre for Nurses and Midwives in Krosno.

It was estimated that 1064 nurses should be included in the study based on the size of the nursing population in Poland (n = 288,395) (Naczelna Izba Pielęgniarek i Położnych, 2017) (fraction size = 0.5; level of confidence = 95%; maximum error = 3%).

All nurses were informed about the aim and character of the research, as well as about how the obtained results would be used. All participants consciously and voluntarily expressed their consent to participate in the research. The research comprised nurses who had a diploma in professional nursing and who had been exercising their profession for at least a year. Pregnant women and people who had been exercising their profession for less than a year, as well as the ones who did not express their voluntary consent, were excluded from the research.

The Bioethics Committee of the Medical University of Silesia approved the research (KNW 0022/KB/89/1/17). All participants expressed their consent for their inclusion in the research through returning the completed, anonymous questionnaires.

### 2.2. Variables and Instruments

After meeting the above criteria, a total of 1200 participants received the questionnaire in an envelope. After completing the questionnaire, the respondents returned them to the researcher. In total, 1080 nurses returned the completed questionnaire (which means that the percentage of responses amounted to 90%).

The questionnaire contained questions concerning demographic data (age, sex, length of service, type of ward, marital status, additional employment).

It also contained the Polish version of the AUDIT-C test (The Alcohol Use Disorders Identification Test Consumption; https://auditscreen.org, accessed on 13 March 2017) for the identification of alcohol addiction [18]. This is a short screening test serving for the detection of an alcohol-related problem. AUDIT-C consists of three questions. The norm for women amounts to 3 points, and for men the norm is 4 points. Exceeding the above values may reflect the occurrence of an alcohol-related problem—harmful drinking or addiction [18,19,20].

The Bielińska test was applied to evaluate diet. The aforementioned scale evaluates nutrition—understood as the frequency and regularity of eating meals and the frequency of the application of food products, being the sources of specific nutrients. The evaluation is made based on the points awarded. The higher the result, the better the means of nutrition. A score of 31 or more points means the person uses an appropriate menu; however, obtaining a lower result is an indicator for necessary improvement [21] (Cronbach’s alpha of 0.77).

A shortened version of the International Physical Activity Questionnaire (IPAQ) was also applied [22]. The information concerns time spent sitting and walking, and time devoted to physical activity, both intensive and moderate. Insufficient physical activity was defined as <600 MET min/week; sufficient physical activity was defined as between 600 and 1500 MET min/week; and high physical activity > 1500 MET min/week, with intensive effort at least 3 days a week or at least 3000 MET min/week [23]. Although the repeatability of the IPAQ was low in this study (Cronbach’s alpha of 0.43), high Spearman’s rank correlations were still observed among the seven variables, indicating that the quality of this survey was maintained.

The Psychosocial Working Conditions (PWP) [24] questionnaire was used to measure wellbeing, as well as two factors related to physical (D1; Cronbach’s alpha of 0.80) and mental mood (D2; Cronbach’s alpha of 0.85), which are jointly described as psychophysical mood (Cronbach’s alpha of 0.86). High values reflect a higher level of wellbeing, and the questionnaire is equipped with norms for nurses.

We also applied the Brief-COPE questionnaire [25] to evaluate the typical ways of reacting and experiencing stressful situations (the Polish adaptation by Z. Juczyński and N. Ogińska-Bulik). Brief-COPE consists of 28 items, comprising 14 strategies of stress divided into 7 groups: active management, acceptance, sense of humour, turning to religion, searching for support, avoidance, helplessness (split-half reliability of 0.72, Guttman index of 0.81). The respondents evaluate each item on a scale from 0 (“I haven’t been doing this at all”) to 3 (“I’ve been doing this a lot”) [26].

The Framingham Type A Scale, by Chesney and Rosenman, was applied to evaluate type A behaviour patterns, in the Polish adaptation by Z. Juczyński [27]. The tool evaluates the intensity of 2 characteristic features for Type A behaviour pattern: haste and competition. Type A scale contains 10 items, out of which the first 5 concern the features and properties typical for the unit, the next 4 concern feelings at the end of a usual day, and the last concerns time pressure. The general result of the type A scale, similarly to both factors, is within the range of 0–1. The results approximating a value of “1” indicate the sense of time pressure and the tendency to compete; however, the ones approximating the value of “0” reflect the dominance of type B behaviours. (Cronbach’s alpha of 0.66). In group research, the classification of A, B, and intermediate behaviours can be performed. This classification, in the absence of normative data, is based on the values of the standard deviation (SD) and the mean (M).

### 2.3. Statistical Analysis

The chi-squared independence test was applied to evaluate the correlations between nominal variables (type A/B behaviour patterns and age, marital status, additional employment, type of ward, attitude to smoking, alcohol consumption, physical activity). Some risk factors were compared using the Kruskal–Wallis analysis of variance (ANOVA) test. All statistical tests were calculated at the significance level alpha ≤ 0.05. The programs used were SPSS and Statistica.

## 3. Results

### 3.1. Characteristics of the Studied Group

In total 1080 nurses aged 24–63 years (average age of 42.8 years) were the subject of this analysis. From the studied group, 378 (35%) nurses consumed alcohol in a harmful way, and one-fifth of them smoked cigarettes. As many as 848 (80.5%) achieved a sufficient level of physical activity (>600 MET), and for the remaining 206 (19.5%), this level was too low (below 600 MET). However, the menus of almost all nurses (n = 1021; 94.5%) required an improvement. Regarding psychophysical wellbeing, resulting from the experienced stress, over half (n = 735; 68%) of the studied group obtained an average result and 179 (16.6%) obtained a low result. The general description of the studied group is summarized in Table 1.

### 3.2. The Prevalence of Type A/B Behaviour Patterns among Polish Nurses

Type A behaviour pattern was demonstrated by 1/3 of the studied nurses and it most frequently occurred with those after the age of 50 (n = 56, 36.6%) and between the age of 31 and 40 (n = 75; 36.1%). The significantly more frequent occurrence of type A behaviour pattern was observed in the group of nurses with additional employment.

The nurses with type A behaviour pattern (mean 3.76 [0.50]) had a significantly better mental and physical mood compared with the nurses demonstrating moderate (mean 3.60 [0.46]) and type B behaviour patterns (mean 3.29 [0.52]) (on PWP scale) (*p* < 0.001; Kruskal–Wallis ANOVA). The nurses characterized by type A behaviour patterns had on average higher results in the range of strategies belonging to the groups: active management, searching for support, acceptance, turning to religion, and sense of humour, compared with the remaining behaviour patterns.

The nurses representing type A behaviour patterns had a significantly smaller risk of alcohol-related problems (*p* = 0.003, chi-square test) compared with the remaining behaviour patterns. In addition, the people characterized by type A behaviour patterns had significantly better eating habits (*Bielińska’s* test for diet); *p* = 0.016; Kruskal–Wallis ANOVA) compared with people with type B behaviour patterns (Table 2).

## 4. Discussion

Type A behaviour pattern is often defined as “a risk factor”, meaning that the probability of the occurrence of disease increases with the intensity of the characteristic features for the above pattern. It is not equivalent, however, with the effect of the impact of many factors, including behaviours unfavourable for health, which occur because of the mediation of the pattern discussed [29].

Pursuant to the results achieved from the conducted research, the type A behaviour pattern was observed among a third of the respondents and it concerned older nurses (after 30 years old) more often. A type A behaviour pattern results from the interactions of individual personality predispositions, the specificity of the environment, and the surroundings [30]. The work of a contemporary nurse imposes strict requirements on the timing, accuracy, and diversity of the tasks conducted [31]. The research proves that the compulsion for fast performance of tasks causes employees to be less patient and more stressed, and consequently to be prone to type A behaviour patterns [30]. Additionally, in the research by G. Nowicki et al. [32], age differentiated people in terms of the incidence of type A behaviour patterns. The most exposed features of the pattern discussed were observed in the age range of 31–50 years old.

The significantly more frequent occurrence of type A behaviour patterns in the group of nurses with additional employment was indicated in our research. Additional employment requires living in constant haste and constitutes a challenge in reconciling professional and personal life. A chronic sense of a lack of time is accompanied by a lack of relaxation, impatience, and acceleration of most of the actions conducted. As a result, it may have an impact on irritability and increase potential anger and hostility, which are concealed among people with type A behaviour patterns [30]. In addition, people with type A behaviour patterns cannot resign from professional achievements, they demonstrate their high need for dominance and control [33]. Thus, the described behaviour pattern may be perceived as adaptive, positively influencing work results, e.g., education results [34]; however, this comes with a lack of control in coping with stress [35]. The people characterised by type A behaviour had, on average, higher results within the strategies belonging to the group: active management, searching for support, acceptance, turning to religion, and sense of humour. In accordance with the available literature, the people with type A behaviour may react to stress by mobilization, and they may take actions aimed at coping. However, in the situation of losing control and inability to regain it, their reactions may take the form of helplessness and hopelessness [29]. The research by D. Włodarczyk and A. Pawliszewska demonstrated a complex image of dependence between the components of Type A behaviour patterns and the professional functioning of nurses. The above research confirmed the predictive value of the content elements of type A behaviour patterns for professional burnout and professional satisfaction. It was indicated that the tendency of nurses for aggression exerts a negative influence; however, the need for domination and achievements is related to better professional functioning. The effect of experiencing stress at work is related to the ability to cope with it [36].

The results of the conducted research indicate that the people presenting type A behaviour patterns also had a better psychophysical mood compared with other behaviour patterns (that is, intermediate type and type B). The obtained result is quite a big surprise because, according to the available literature, the risk taken and constant willingness to exercise control over their surroundings causes people with a type A behaviour patterns to be more prone to stress [37,38]. However, no correspondence was found between a psychophysical mood and type A behaviour pattern in a recent study [10]. On the other hand, an interesting study was conducted in China among medical staff working in the COVID-19 pandemic. Medics were exposed to severe stress. The results of the mediation analysis demonstrated that depression significantly mediated the relationship between type A personality, appetite, and sleep disorders [39]. This proves that it is still difficult to identify a clear-cut model explaining the relation between type A behaviour patterns and psychophysical mood.

Additionally, this study indicated that the risk of alcohol-related problems affected people with type B behaviour patterns more often compared with type A. However, the nurses characterized as type A had significantly better eating habits.

The research by G. Nowicki et al. [32], conducted on 150 working people, indicates that some variables, including appropriate eating habits, cholesterol concentration, and arterial tension, explain 54% of variance related to type A behaviour patterns. The analysis proves that the intensity of the features of type A behaviour patterns increases with the increase in appropriate eating habits and the level of cholesterol concentration in blood serum. On the other hand, in the research by A. Rogowska [40], the students demonstrating type A behaviour patterns presented statistically significantly “worse” health behaviours compared with the students with type B. In the quoted research, the scale of alcohol consumption did not indicate a statistically significant correlation with type A behaviour pattern. However, the research by N. Ogińska-Bulik and J. Chanduszko-Salska [41] did not confirm the relation between type A behaviour patterns and the risk factors of cardiovascular diseases, such as smoking, arterial hypertension, or increased cholesterol concentration in blood serum. A similar lack of association between the prevalence of hypertension and type A personality was observed in a recent study in Japan [42]. The authors suggested that this was a result of the existence of strong social support systems in Japanese culture.

Regarding the present study, type A behaviour patterns must be regarded as a significant factor differentiating certain lifestyle aspects among Polish nurses.

Regarding the growing demand for care, and the ageing population of nursing staff, we must consider the implementation of strategies aiming to improve their mental and physical health. Keeping the above in mind, managers of nurses should implement strategies aimed at the evaluation of wellbeing and healthy or at-risk behaviours, e.g., by the application of screening tests identifying the staff at risk. Promoting healthy behaviours and developing stress-management techniques among nurses may improve nurses’ wellbeing and professional performance, and help set a better example for patients. The application of consistent measurement tools would enable researchers to compare results from various studies, drawing relevant conclusions concerning the improvement of nurses’ health; this measure is advisable.

## 5. Limitations

Our study had certain limitations. The data collected in this research were based on voluntary questionnaires conducted in one region in Poland; therefore, the study may not reflect all nurses working in all regions of Poland. We included, however, a big population (N = 1080), which additionally convinces us that the data are representative of the national population of nurses in Poland. In the research, we applied measurement tools based on self-reporting, which is related to the possibility of the occurrence of the social approval bias—the willingness of the respondents to present themselves in a better light. In addition, the research had a cross-sectional character. We cannot unequivocally state, based on it, any cause-and-effect relations.

## 6. Conclusions

The occurrence of a “coronary prone behaviour pattern” was found to be related to health-promoting behaviours among nurses, that is, a better mental and physical mood, better ability to cope with stress, a smaller risk of alcohol-related problems, and proper nutrition. There is a need for further research on the dependence between type A behaviour patterns and lifestyle.

## Figures and Tables

**Table 1:** General characteristics of the analysed group of nurses in Poland [28].

Characteristic	N (%)
Sex	
Male	28 (2.6%)
Female	1052 (97.4%)
Age (years)	
≤30	132 (12.3%)
31–40	208 (19.2%)
41–50	587 (54.3%)
≥51	153 (14.2%)
Marital status	
Single	760 (71.1%)
Married	105 (9.8%)
Divorced	178 (16.7%)
Widowed	26 (2.4%)
Additional employment	
Yes	397 (36.8%)
No	638 (63.2%)
Type of ward	
Surgical	354 (32.7%)
Non-surgical	726 (67.3%)
Attitude to smoking	
Smoker	214 (19.8%)
Non-smoker	866 (80.2%)
Alcohol consumption ^‡^	
Risk of alcohol addiction	378 (35%)
No risk of alcohol addiction	702 (65%)
Physical activity ^#^	
Insufficient (<600 MET min/week)	206 (19.5%)
Sufficient (600–1500 MET min/week)	315 (29.9%)
High (>1500 MET min/week)	533 (50.6%)
Psychophysical mood ^¶^	
High	166 (15.4%)
Average	735 (68%)
Low	179 (16.6%)
Eating habits ^⁜^	
Normal diet	59 (5.5%)
Poor diet	1021 (94.5%)
Brief-COPE *	M (SD)
Active management	
Active management	1.61 (0.71)
Planning	2.09 (0.65)
Positive assessment	1.88 (0.70)
Acceptance	1.82 (0.68)
Sense of humor	0.95 (0.66)
Turning to religion	1.25 (0.98)
Searching for support	
Emotional support	1.87 (0.75)
Instrumental support	1.81 (0.74)
Avoidance	
Dealing with something else	1.62 (0.70)
Denial	0.94 (0.72)
Abreaction	1.34 (0.66)
Helplessness	
Taking psychoactive substances	0.33 (0.60)
Withdrawal from activities	0.88 (0.67)
Blaming oneself	1.26 (0.76)

Abbreviation: M (SD)—mean (standard deviation). ^‡^ Based on the AUDIT-C screening test for risk of alcohol abuse. ^#^ Based on the IPAQ International Physical Activity Questionnaire. ^¶^ Based on the Psychosocial Working Conditions (PWP) questionnaire. ^⁜^ Based on the Bielińska’s test for diet. * Brief-COPE: Scale from 0 (“I haven’t been doing this at all”) to 3 (“I’ve been doing this a lot”).

**Table 2:** Characteristics of an analysed group of nurses including the occurrence of type A/B behaviour patterns and health behaviours.

Parameter	Type A Personality (n = 333; 30.8%) N (%)	Type Intermediate (n = 475; 44%) N (%)	Type B Personality (n = 272; 25.2%) N (%)	*p*-Value (Chi-Squared Test)
Age (years)				0.024
≤30	31 (23.5%)	58 (43.9%)	43 (32.6%)	
31–40	75 (36.1%)	93 (44.7%)	40 (19.2%)	
41–50	171 (29.1%)	268 (45.7%)	148 (25.2%)	
≥51	56 (36.6%)	56 (36.6%)	41 (26.8%)	
Marital status				0.141
Single	51 (28.7%)	74 (41.6%)	53 (29.8%)	
Married	238 (31.3%)	348 (45.8%)	174 (22.9%)	
Divorced	35 (33.3%)	42 (40.0%)	28 (26.7%)	
Widowed	8 (30.8%)	7 (26.9%)	11 (42.3%)	
Additional employment				0.044
Yes	138 (34.8%)	173 (43.6%)	86 (21.7%)	
No	195 (28.6%)	302 (44.2%)	186 (27.2%)	
Type of ward				0.291
Surgical	94 (26.6%)	162 (45.8%)	98 (27.7%)	
Non-surgical	178 (24.5%)	313 (43.1%)	235 (32.4%)	
Attitude to smoking				0.811
Smoker	64 (19.2%)	94 (19.8%)	56 (20.6%)	
Non-smoker	269 (80.8%)	381 (80.2%)	216 (79.4%)	
Alcohol consumption ^‡^				0.003
Risk of alcohol addiction	92 (27.6%)	183 (38.5%)	103 (37.9%)	
No risk of alcohol addiction	169 (72.4%)	292 (61.5%)	169 (62.1%)	
Physical activity ^#^				0.289
Insufficient	54 (17.0%)	89 (19.0%)	63 (24.0%)	
Sufficient	97 (30.0%)	139 (30.0%)	79 (30.0%)	
High	169 (53.0%)	241 (51.0%)	123 (51.0%)	
	**M (SD)**			***p***-**Value** **(Kruskal-Wallis ANOVA)**
Eating habits ^⁜^	16.66 (6.11)	16.30 (5.52)	15.49 (6.52)	0.016
Psychophysical mood ^¶^	3.76 (0.50)	3.60 (0.46)	3.29 (0.52)	<0.001
Active management *				
Active management	1.83 (0.78)	1.59 (0.64)	1.35 (0.64)	<0.001
Planning	2.40 (0.66)	2.08 (0.59)	1.72 (0.55)	<0.001
Positive assessment	2.16 (0.75)	1.88 (0.58)	1.51 (0.66)	<0.001
Acceptance *	2.04 (0.79)	1.79 (0.61)	1.61 (0.57)	<0.001
Sense of humor *	1.09 (0.74)	0.93 (0.60)	0.82 (0.60)	<0.001
Turning to religion *	1.40 (1.08)	1.19 (0.95)	1.18 (0.89)	0.016
Searching for support *				
Emotional support	2.01 (0.79)	1.90 (0.70)	1.63 (0.72)	<0.001
Instrumental support	1.94 (0.83)	1.84 (0.66)	1.60 (0.70)	<0.001
Avoidance *				
Dealing with something else	1.68 (0.79)	1.62 (0.66)	1.55 (0.64)	0.066
Denial	0.83 (0.73)	0.91 (0.69)	1.12 (0.72)	<0.001
Abreaction	1.26 (0.71)	1.34 (0.63)	1.44 (0.62)	0.003
Helplessness *				
Taking psychoactive substances	0.18 (0.44)	0.33 (0.57)	0.51 (0.74)	<0.001
Withdrawal from activities	0.59 (0.70)	0.90 (0.62)	1.19 (0.57)	<0.001
Blaming oneself	1.02 (0.77)	1.23 (0.68)	1.62 (0.73)	<0.001

Abbreviation: M (SD)—mean (standard deviation). ^‡^ Based on the AUDIT-C screening test for risk of alcohol abuse. ^#^ Based on the IPAQ International Physical Activity Questionnaire. ^¶^ Based on the Psychosocial Working Conditions (PWP) questionnaire. ^⁜^ Based on *Bielińska’s* test for diet. * Brief-COPE: Scale from 0 (“I haven’t been doing this at all”) to 3 (“I’ve been doing this a lot”).

## Data Availability

The data could be obtained from the corresponding author on reasonable request.
